# Deciphering the Molecular Mechanism of Escin against Neuropathic Pain: A Network Pharmacology Study

**DOI:** 10.1155/2023/3734861

**Published:** 2023-10-16

**Authors:** Xi Li, Yating Wu, Haoyan Wang, Zaiqi Li, Xian Ding, Chongyang Dou, Lin Hu, Guizhi Du, Guihua Wei

**Affiliations:** ^1^School of Life Science and Engineering, Southwest Jiaotong University, Chengdu, China; ^2^Department of Anesthesiology, Laboratory of Anesthesia and Critical Care Medicine, National-Local Joint Engineering Research Centre of Translational Medicine of Anesthesiology, West China Hospital, Sichuan University, Chengdu, China

## Abstract

**Background:**

Escin is the main active component in *Aesculus hippocastanum*. It has been demonstrated that escin has anti-inflammatory properties. This study combined the methods of network pharmacology, molecular docking, and molecular dynamics to explore the molecular mechanism of escin against neuropathic pain (NP).

**Methods:**

The Swiss Target Prediction and the Pharm Mapper database were employed for predicting the targets of escin. Also, the candidate targets of NP were gathered via the databases including Therapeutic Targets, DisGeNet, GeneCards, DrugBank, and OMIM. Subsequently, the network of protein-protein interaction was screened for the key targets by the software Cytoscape 3.8.0. Then, the intersection of these targets was analysed by Gene Ontology (GO) and Kyoto Encyclopedia of Genes and Genomes (KEGG) enrichment. Additionally, we further investigated the ligand-target interactions by molecular docking and molecular dynamics simulations.

**Results:**

In total, 94 escin targets were predicted by network pharmacology. Among them, SRC, MMP9, PTGS2, and MAPK1 were the core candidate targets. Subsequently, the analysis of GO and KEGG enrichment revealed that escin affected NP by regulating protein kinase C, MAP kinase, TRP channels, the T-cell receptors signaling pathway, and the TNF signaling pathway. The results of molecular docking and molecular dynamics simulation confirmed that escin not only had a strong binding activity with the four core target proteins but also stably combined in 50 ns.

**Conclusions:**

Our study revealed that escin acts on the core targets SRC, MMP9, PTGS2, MAPK1, and associated enrichment pathways to alleviate neuronal inflammation and regulate the immune response, thus exerting anti-NP efficacy. This study provided innovative ideas and methods for the promising treatment of escin in relieving NP.

## 1. Introduction

The International Association for the Study of Pain defines neuropathic pain (NP) as pain caused by a lesion or disease of the somatosensory nervous system [1]. Prevalence rates of NP vary between 11% and 40% in the USA [2]. Also, NP is frequently accompanied by sensory abnormalities, such as allodynia, numbness, and pain paroxysms, while the duration varied from months to years [3]. The mechanisms of NP are complex, and the therapies are poorly efficacious [4]. Injury, trauma, infection, and inflammation of the central or peripheral nervous system lead to NP. Recent studies have found that neuropathic pain was often accompanied by the release of inflammatory cytokines and chemokines that were mediated by neurons, glial cells, and large numbers of immune cells in the nervous system. Inflammatory mediators and various cytokines play an important role in neuronal inflammation and the sensitivity of primary afferent neurons to pain [5]. However, the complex pathological mechanisms underlying the inflammation of NP remains are still unclear [6]. Little therapies have been found in the last decade years, except for antidepressants, antiepileptics, and opioid analgesics, which have their different problems of hepatotoxicity, cardiovascular disorders, central nervous system disturbances, etc. [7].

Lots of studies reported that natural compounds compared with synthetic compounds had the characteristics of being valid, nontoxic, safe, cheap, etc. [8–10]. Thus, studies on natural anti-NP drugs are essential for improving NP treatment and prognosis. Escin, the main active component of *Aesculus hippocastanum* seed, has attracted attention owing to its anti-inflammatory effects [11]. Escin also possesses the capacities of antioedematous [12], anticancer [13], antioxidant effects [14], etc. This study reported that escin significantly decreased the NF-kappa B expression of lipopolysaccharide mice for its anti-inflammatory effect [15]. Moreover, Cheng et al. found that escin reduced the concentration of the proinflammatory cytokines TNF-*α*, IL-1*β*, and IL-6 in endotoxemia mice and LPS-induced macrophages [16]. Numerous studies have shown that inhibiting the proinflammatory cytokines and chemokines in the dorsal root ganglia, spinal cord, and brain would relieve NP [6]. Therefore, we speculated that escin with the anti-NP character would alleviate NP by relieving the neuroinflammation in the peripheral nervous system and central nervous system.

Network pharmacology that integrates bioinformatics and multivariate pharmacology analyses the connection between natural compounds and the key targets protein, and it is a rapid and convenient new approach [17]. In addition, for natural compounds, network pharmacology which has the advantages of “multitarget and multipathway” provides the feasible and theoretical method for exploring the mechanism in NP [18]. For the interactions of ligand-target, we employed molecular docking and molecular dynamics simulations to verify the results of network pharmacology [19, 20].

**Figure 1:**
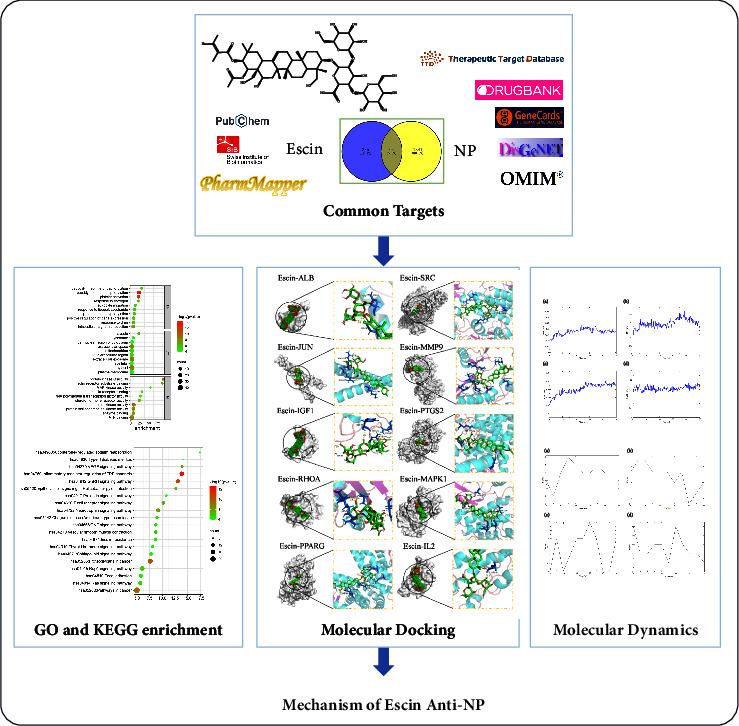
The process of exploring the molecular mechanism of escin against NP.

In this article, combining network pharmacology and molecular docking with molecular dynamics simulation, we investigated the potential targets and signaling pathways of escin in treating NP. This study might provide a new combination of escin for the treatment of NP. The process drawing is shown in Figure 1.

## 2. Materials and Methods

### 2.1. Potential Target Prediction of Escin

The mol2 format file and canonical SMILEs of escin were obtained from the PubChem database (https://pubchem.ncbi.nlm.nih.gov/). The Swiss Target Prediction database (https://www.swisstargetprediction.ch/) was used for predicting the potential targets of bioactive compounds. The canonical SMILEs were imported into Swiss Target Prediction with the criteria of “Homo sapiens” and the probability of ≥0.6. The Pharm Mapper database (https://www.lilab-ecust.cn/pharmmapper/) was used for the method reverse pharmacophore matching to retrieve the targets of compounds, and the matching results were scored and ranked. The mol2 format file of escin was uploaded to Pharm Mapper for target prediction with the criteria of “Homo sapiens” and normal fit score ≥0.3 [21, 22]. Then, the intersections of the two analyses were input into the UniProt database (https://www.uniprot.org/) to normalize the gene names.

### 2.2. Target Genes Collection for Neuropathic Pain

NP-associated target genes were collected through the databases, including Therapeutic Targets Database (https://db.idrblab.net/ttd/), DisGeNet (https://www.disgenet.org/), GeneCards (https://www.genecards.org), DrugBank (https://www.drugbank.ca), and OMIM (https://omim.org/). After discarding the duplicate targets, we obtained NP-related targets [23].

### 2.3. Construction of Protein-Protein Interaction (PPI) Network

By comparing the intersection target genes of escin and NP, we need to obtain the potential targets of escin for alleviating NP by the PPI network [24]. We set the criteria by choosing the confidence score of ≥0.4 in Homo sapiens and obtained the PPI network through the STRING database (https://www.string-db.org/). Also, the results were exported as “TSV” format files. Then, the TSV file was imported into Cytoscape 3.8.2 software. After topology analysis by “Network Analyzer,” we mapped the network of common target interactions and calculated the values of node degree. According to the node degree values of the targets, we obtained the top 10 key targets for escin anti-NP [25].

### 2.4. Analysing Gene Ontology (GO) and Kyoto Encyclopedia of Genes and Genomes (KEGG) Pathway Enrichment

GO and KEGG enrichment analyses of target genes were employed through DAVID (https://david.ncifcrf.gov/). Also, biological process (BP), cellular component (CC), and molecular function (MF) are the three parts of GO enrichment. To determine the biological significance of the target genes, we set the criteria by choosing the probability score ≤0.05 as the cutoff values and obtained the top −log_10_ (P) terms in the results.

### 2.5. Molecular Docking

Molecular docking was used to predict the patterns of docking and binding affinity between escin and the top 10 targets in 3D structures. The crystal structures of the top 10 targets were downloaded from the RCSB Protein Data Bank database (https://www.rcsb.org/) and saved in PDB format. After removing water molecules and residual ligands by PyMol-2.4.0 and adding hydrogen in AutoDock (version 1.5.6), we obtained the optimized structure of the top 10 targets. After molecular docking by AutoDock Vina, we obtained receptor-ligand docking energy. Finally, we visualized the escin-target complexes with the lowest binding energy by PyMol-2.4.0 [26].

### 2.6. Molecular Dynamics Simulation

The escin-target complex interactions were performed by molecular dynamics simulation with the GROMACS 2020.6 software and the CHARMM36 force field [27, 28]. The four lowest binding energy complexes were carried out with the following methods. The temperatures and pressure were 255 K and 40 Pa (freeze-drying) and 300 K and 100000 Pa (spray-drying). For each simulation, the transferable intermolecular potential 3 points (TIP3P) water model was added to the periodic cubic box for solvation, with the minimum distance between the protein and the box being 1.0 nm. All simulations were performed for 50 ns. A series of MD trajectory analyses such as root mean square deviation (RMSD), root mean square fluctuation (RMSF), and radius of gyration (Rg) were applied to analyse the simulation results to assess the stability of binding in a dynamic environment.

## 3. Results

### 3.1. Potential Targets of Escin in the Treatment of NP

**Figure 2:**
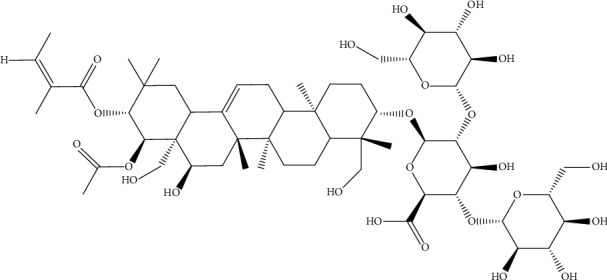
Chemical structure of escin.

**Figure 3:**
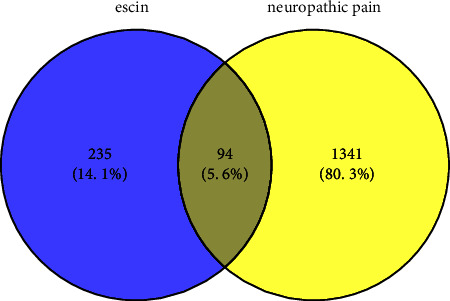
94 common targets between escin and NP.

The chemical structure of escin (Figure 2) obtained from the PubChem database was uploaded to the Swiss Target Prediction and Pharm Mapper platform. After removing the duplicate data, a total of 329 potential targets of escin were identified. Then, the databases of OMIM, DisGeNet, GeneCards, DrugBank, and TTD were used to search for NP-related targets. After merging the targets from these databases and removing duplicate data, a total of 1435 NP-related targets were obtained. The 94 common potential targets between escin and NP were shown in the Venn diagram (Figure 3). Also, detailed information of the target proteins is listed in Table S1.

### 3.2. Construction and Analysis of PPI Network

**Figure 4:**
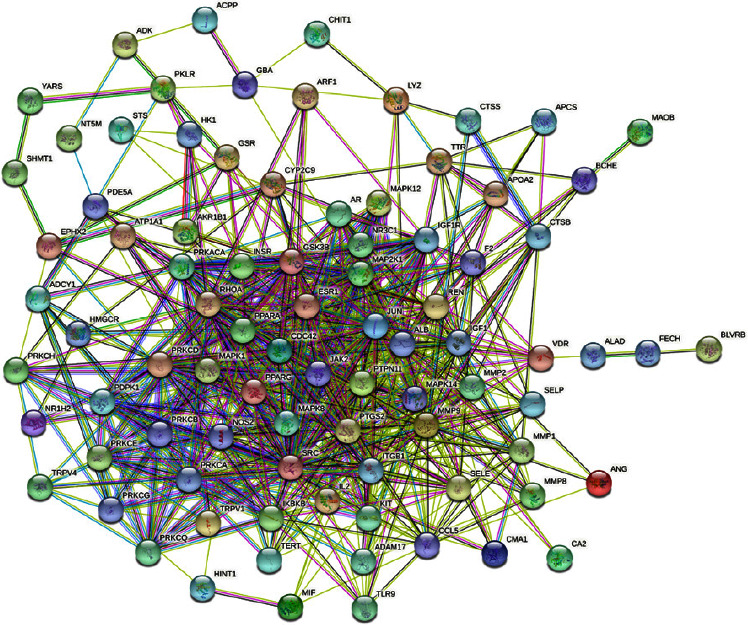
The network of PPI analysis.

**Figure 5:**
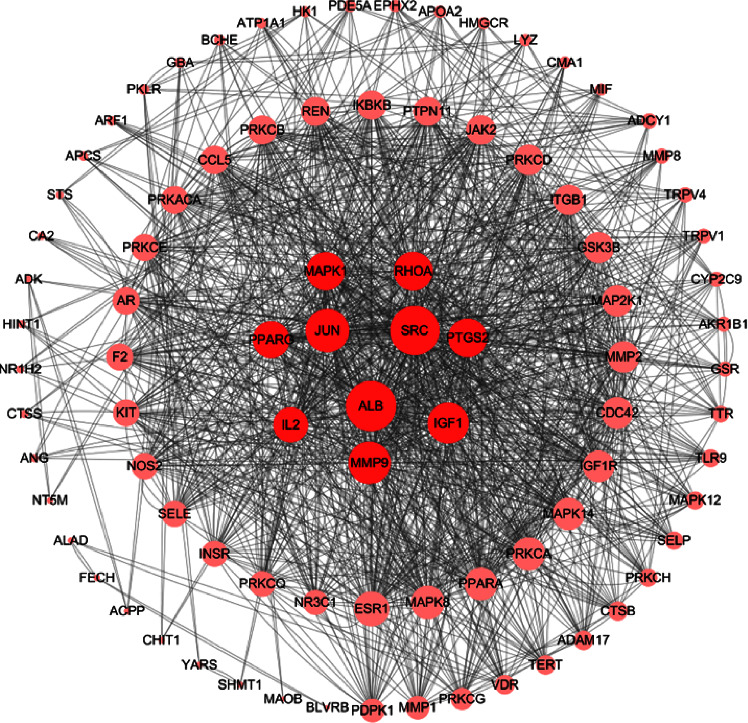
Cytoscape enrichment analysis. The targets used to treat NP were represented by the circular nodes; the larger the node size, the larger its degree value. The red node represents the key targets.

The STRING database and Cytoscape 3.8.2 were used to construct a PPI network of the 92 candidate target proteins. As shown in Figure 4, there were 92 nodes (target proteins) and 652 edges (the interactions between proteins) in the PPI network. After screening by “Network Analyzer,” we obtained the top ten highest-degree targets in the network, which are shown in red (Figure 5). The results of PPI indicated that ALB, SRC, JUN, MMP9, IGF1, PTGS2, RHOA, MAPK1, PPARG, and IL2 were the key targets for the efficacy of escin (Table 1).

### 3.3. GO and KEGG Pathway Enrichment Analyses

**Figure 6:**
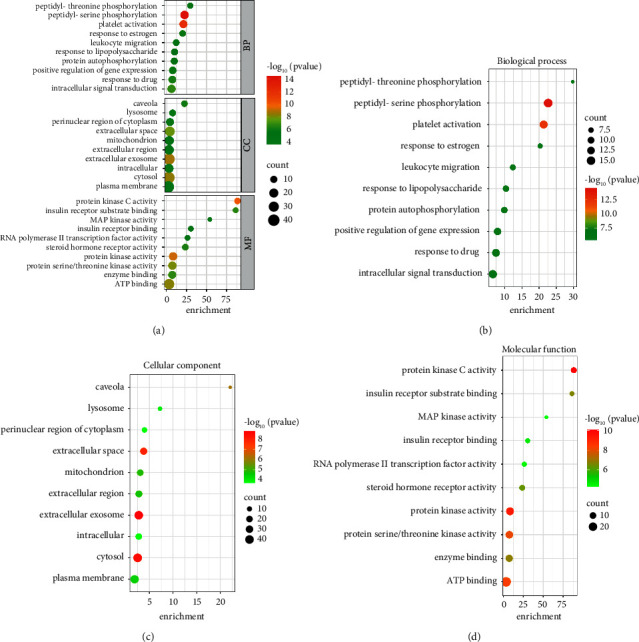
GO enrichment analysis. (a) GO enrichment analysis of candidate targets, (b) the top 10 biological process (BP), (c) the top 10 cellular component (CC), and (d) the top 10 molecular function (MF) of GO enrichment.

**Figure 7:**
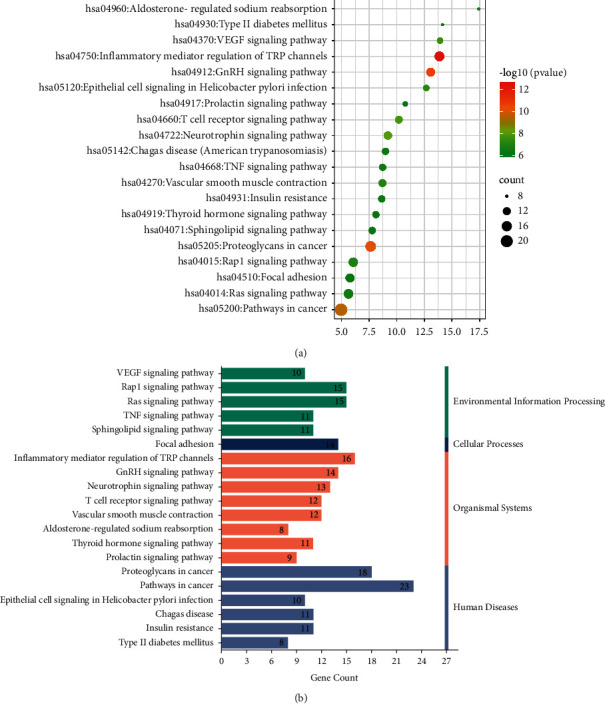
KEGG enrichment analysis. (a) The top 20 KEGG pathways of candidate genes. (b) Classified KEGG pathways of candidate genes.

We performed GO and KEGG pathway enrichment analyses in the DAVID to determine the multiple molecular mechanisms of escin against NP. 272 enriched GO terms were obtained by GO functional enrichment of DAVID, including 186 BP terms, 30 CC terms, and 56 MF terms (Figure 6(a)). BP results determined that these targets provided responses to peptidyl-serine phosphorylation and peptidyl-threonine phosphorylation (Figure 6(b)); CC results indicated that these targets were located in caveolae and lysosome (Figure 6(c)); MF results included protein kinase C activity, insulin receptor substrate binding, MAP kinase activity (Figure 6(d)), and so forth. A total of 103 related pathways linked to escin anti-NP activity were obtained. According to the results, most genes were involved in the top 20 signaling pathways (Table S2, Figure 7(a)). Also, these pathways were classified into four major groups: environmental information processing, cellular processes, organismal systems, and human diseases (Figure 7(b)). It can be concluded that the anti-NP effects of escin were associated with inflammatory mediator regulation of the TRP channel, T-cell receptors, and the TNF signaling pathway. These results of enrichment analysis might provide meaningful insights to reveal the potential mechanism of escin anti-NP.

### 3.4. Molecular Docking

**Figure 8:**
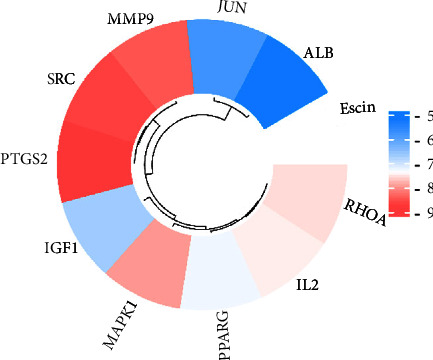
The energy of molecular docking.

**Figure 9:**
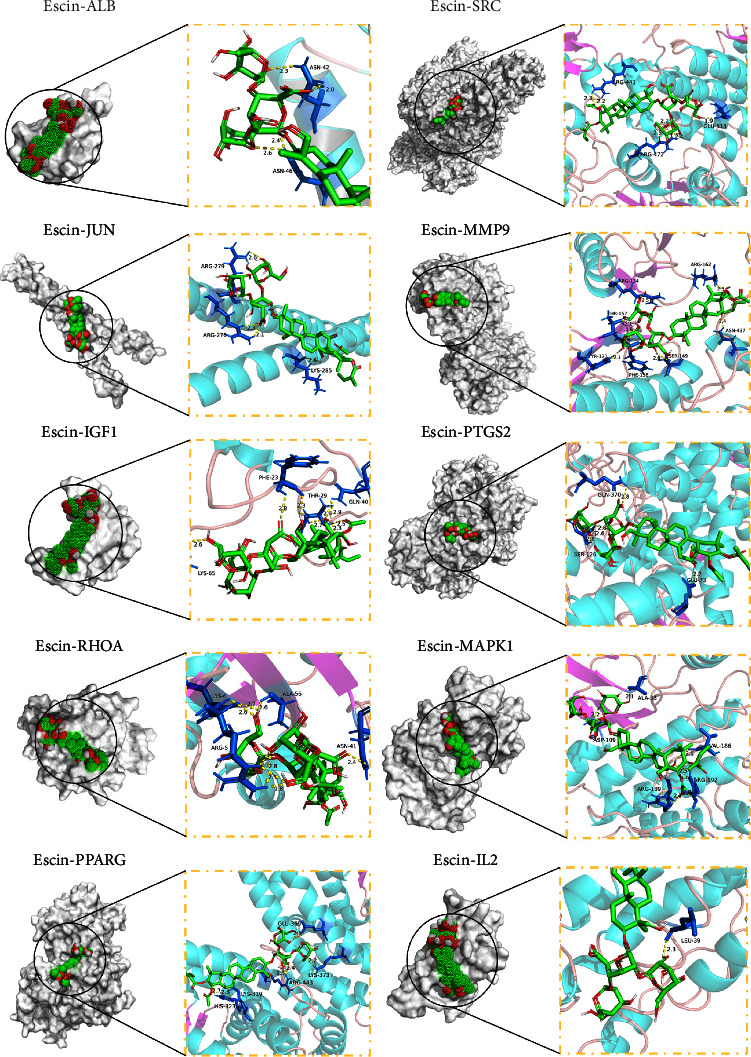
Molecular docking of escin with the top ten targets. Yellow box shows the details.

**Figure 10:**
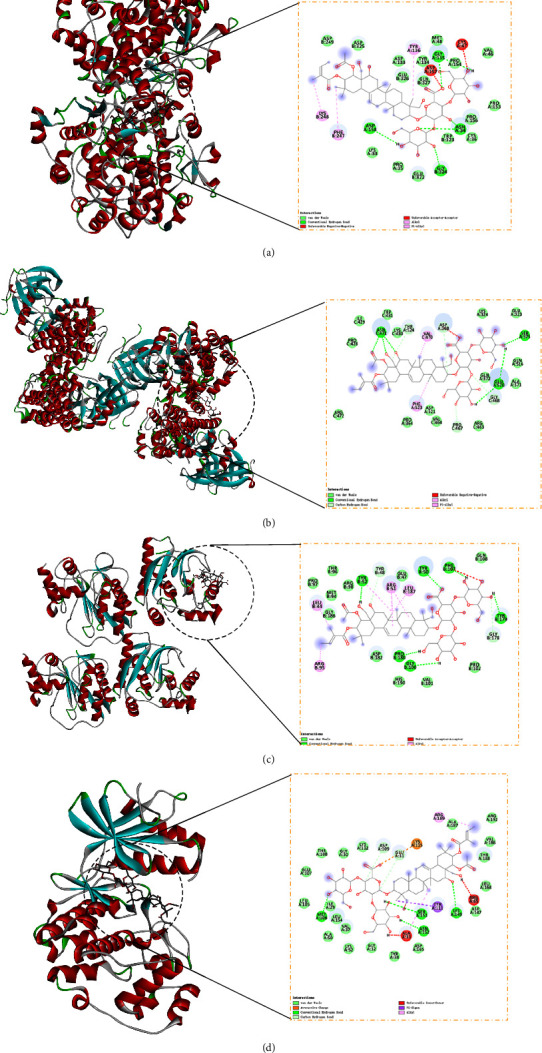
The molecular docking models of escin with four key targets protein. (a) PTGS2; (b) SRC; (c) MMP9; (d) MAPK1.

Molecular docking can validate the association of escin with targets. The docking effect was shown by the binding energy (kcal/mol). Generally, lower binding energy represented better binding between escin and the targets. The binding energy smaller than −5.0 kcal/mol showed good binding activity, whereas the binding energy smaller than −7.0 kcal/mol showed strong binding activity [20, 29]. Our results found that the binding energy of escin and the ten targets was all less than −5 kcal/mol (Figure 8). Their binding patterns mainly involved hydrogen bonding (Figure 9), indicating that the affinity between escin and the key targets was generally good. Notably, escin bound more strongly to PTGS2 (−8.8 kcal/mol), SRC (−8.7 kcal/mol), MMP9 (−8.5 kcal/mol), and MAPK1 (−8.0 kcal/mol) (Figure 10). It has been demonstrated that the four core targets play an important role in neuroinflammation and pain hypersensitivity [30–33]. Thus, we speculated that PTGS2, SRC, MMP9, and MAPK1 may be the core targets of escin against NP. The results suggested that escin acted on the four core target proteins to alleviate NP, and the details of molecular docking are shown in Table 2. For escin and the core targets, there were multiple hydrophobic interactions and hydrogen bonds between molecules and amino acid residues. These outcomes showed the validity of the network pharmacology findings.

### 3.5. Molecular Dynamics Simulation

**Figure 11:**
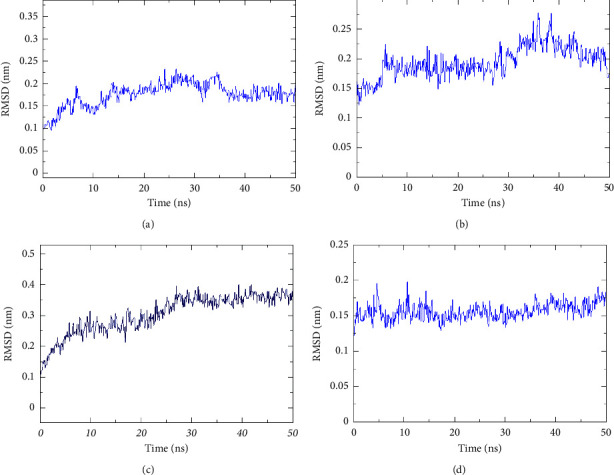
Root mean square deviation (RMSD)-protein plots by molecular dynamics simulation. (a) Escin-PTGS2. (b) Escin-SRC. (c) Escin-MMP9. (d) Escin-MAPK1. The *X*-axis indicates time (ns) and *Y*-axis indicates RMSD (nm).

**Figure 12:**
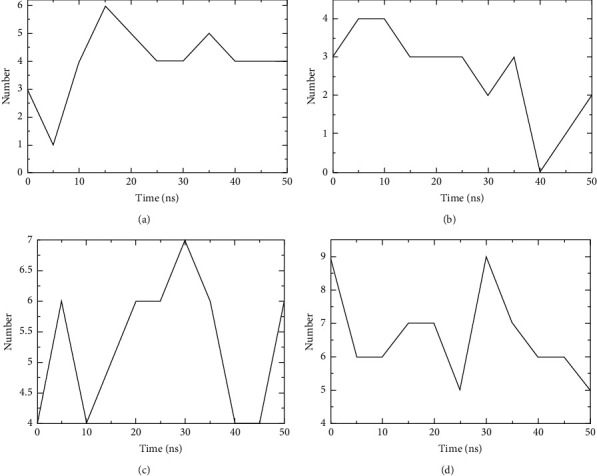
Hydrogen bonds between proteins and ligands by molecular dynamics simulation. (a) Escin-PTGS2. (b) Escin-SRC. (c) Escin-MMP9. (d) Escin-MAPK1. The *X*-axis indicates time (ns), and *Y*-axis indicates the number of hydrogen bonds.

Even though the excellent binding activity of escin to the targets has been confirmed by molecular docking, this approach could not reveal its stability in vivo. Molecular dynamics simulation provided insight into the stability of protein-ligand complexes. The RMSD is typically regarded as a key sign of a system's stability [23]. As shown in Figure 11, the RMSD values of escin with the core targets fluctuated in a small range (RMSD <0.3 nm) during the simulation. The analysis demonstrated that the escin-target complexes possessed stable intermolecular interaction patterns. The hydrogen bond is one of the most vital noncovalent mutual interactions in the binding process of small molecules and proteins. From 10 ns until 30 ns of the simulation, the number of hydrogen bonds formed between escin and protein was maintained at more than 2 (Figure 12). The protein's flexibility during the molecular dynamics simulation could be reflected by the RMSF. The results demonstrated that the amino acid residues showed minor variations during the simulation (<0.4 Å) (Figure S1). The Rg is the root mean square distance of the protein atoms from the axis of rotation [34]. As shown in Figure S2, escin did not cause significant structural alterations in the target proteins. Taken together, the results suggested that escin combined tightly with the core targets SRC, MMP9, PTGS2, and MAPK1 which were in accordance with molecular docking.

## 4. Discussion

NP is caused by damage or disease of the somatosensory system. In the last decade, the prevalence has increased with the development of the global population [1]. The mechanisms of NP are complex and the therapies are poorly efficacious [4]. In this study, we investigated the “multitarget and multipathway” mechanism of escin against neuropathic pain using the methods of network pharmacology, molecular docking, and molecular dynamics. Also, we found that escin anti-NP was predominantly related to neuroinflammatory response and immune regulation. The results showed that 94 escin targets were involved in treating NP. The top 10 targets of the PPI network were identified as the key targets. Among them, SRC, MMP9, PTGS2, and MAPK1 were the core candidate targets. Molecular docking and molecular dynamics simulations verified that escin bound strongly and stably to the four core target proteins in 50 ns. In addition, the results of the escin-target pathway indicated the anti-NP activity of escin may be closely related to the three main signaling pathways, including inflammatory mediator regulation of TRP channels, TNF signaling pathway, and T-cell receptor signaling pathway. Thus, this study provides further insights for exploring the escin anti-NP mechanism.

### 4.1. The Core Targets of Escin Anti-NP

From the 94 intersection targets of PPI results, we further found out the top 10 targets: ALB, SRC, JUN, MMP9, IGF1, PTGS2, RHOA, MAPK1, PPARG, and IL2, which were identified as the key targets by their topological properties. Moreover, the results of molecular docking verified that their binding energy was less than −5 kcal/mol. In Figures 8 and 9, the binding energy of escin with PTGS2, SRC, MMP9, and MAPK1 indicated that they played very important roles in escin anti-NP. Also, there are lots of reports about the four core targets' key effects in neuroinflammation diseases. For example, PTGS2 (−8.8 kcal/mol), the top of four core targets, is involved in the production of prostaglandins via the arachidonic acid pathway. In addition, Zhao et al. demonstrated that PTGS2 induces hypersensitization in NP by expanding the inflammation after spinal cord injury [30]. Also, inhibiting PTGS2 significantly alleviated mechanical hypersensitivity in rats [35]. Similarly, SRC (−8.7 kcal/mol), the second of four core targets, is the critical member of mediating the central and peripheral sensitizations. Inhibiting SRC reduced the activation of microglia which relieved the mechanical nociceptive hypersensitivity of NP after partial sciatic nerve ligation surgery [32, 36]. And MMP-9 (−8.5 kcal/mol), the third of four core targets [37], played an essential role in pathophysiological functions [38]. Kawasaki showed that MMP-9 was associated with the development of NP, and inhibiting MMP-9 relieved the inflammation of NP [32]. Furthermore, procyanidins alleviated the behaviors of chronic constriction of sciatic nerve injury mice by inhibiting the activity of MMP-9/2 [39]. MAPK1 (–8.0 kcal/mol) is also known as extracellular signal-regulated kinase (ERK) 1. Genovese et al. demonstrated that inhibition of MAPK-related signaling pathways may be effective in inflammation after nerve injury [40]. Also, activating MAPK1 in the formalin model is essential in central sensitization [33, 35]. Moreover, our results of molecular dynamics simulations for 50 ns showed the four complexes had stable binding abilities during the simulation. Thus, consistent with these studies of PTGS2, SRC, MMP9, and MAPK1, our results further indicated that escin may significantly alleviate NP by the four core targets, while the mechanism of the four core targets involved needs detailed experiments later.

### 4.2. The Three Signaling Pathways of Escin Anti-NP

**Figure 13:**
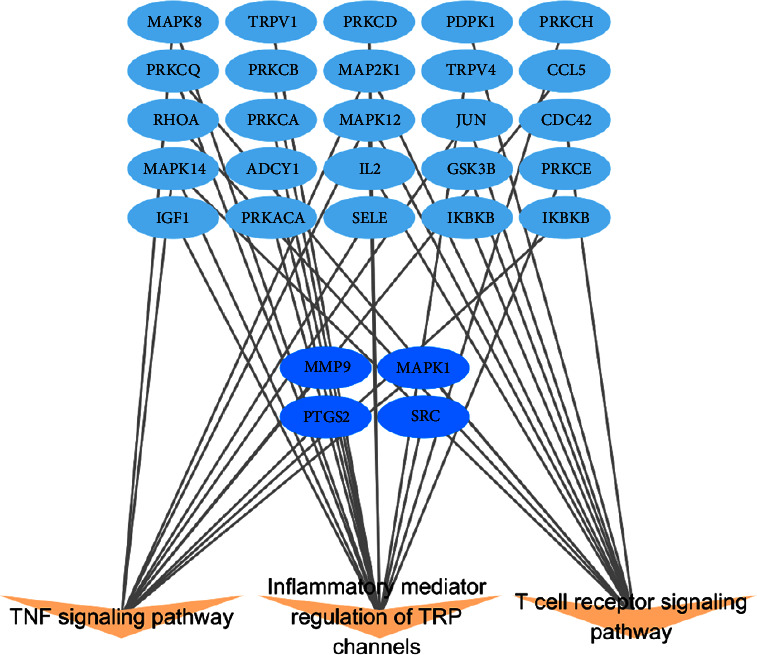
The network of “targets-signaling pathways” of escin against NP. The deep blue nodes represent the four core targets.

Our KEGG enrichment analysis showed that the anti-NP effect of escin may be closely related to inflammatory mediator regulation of TRP channels signaling pathway, TNF signaling pathway, and T-cell receptor signaling pathway. Also, the four core targets (PTGS2, SRC, MMP9, and MAPK1) of our PPI results play critical roles in the above three pathways (Figure 13). The first of three signaling pathways, transient receptor potential (TRP) channels, is often activated and marked by a state of hypersensitivity and hyperexcitability of nociceptors of peripheral sensitization [36]. Our result also demonstrated that escin regulated the inflammatory mediator regulation of TRP channels signaling pathway in treating NP. Also, TRPV1 antagonists could alleviate pain in various nerve injury models [41, 42]. TRP, as a core element of NP, gave a new potential target for treating NP. The second of three signaling pathways, TNF signaling pathway, played an important role in the development of inflammation. Also, our results of the core targets showed that MAPK1, PTGS2, and MMP9 were involved in the TNF signaling pathway in neuroinflammation of NP. Lu et al. demonstrated that TNF-*α* antagonists increased the paw withdrawal thresholds by influencing nerve growth factor [43, 44]. In addition, TNF signaling pathway enhanced the proliferative capacity of T lymphocytes, which mediated the adaptive immune response in NP [45]. The third signaling pathway, T-cell receptor signaling pathway, has an intimate correlation with the incidence of nociceptive hypersensitivity [46]. Also, Kobayashid indicated that T cells migrated significantly after the ligation of the sciatic nerve in mice. Administration of anti-T-cell agents inhibited PSL-induced thermal hyperalgesia and tactile hypersensitivity [47].

In summary, this study demonstrated the anti-NP effect and mechanism of escin by combining network pharmacology with molecular docking and molecular dynamics validation. These results not only provided evidence for guiding the clinical application of escin but also explored the possible molecular mechanisms in the development of NP. Briefly, we found out that escin may act on targets such as PTGS2, SRC, MMP9, and MAPK1 and then regulate TNF, TRP channels, and T-cell receptor signaling pathway, thus alleviating neuroinflammation and against NP (Figure 13).

## 5. Conclusions

Overall, our study provides new ideas that escin has “multitarget and multipathway” anti-NP activity. Escin may act on PTGS2, SRC, MMP9, and MAPK1 and then regulate TNF, TRP channels, and T-cell receptor signaling pathway. These results lay a foundation for investigating the anti-NP mechanism of escin. However, the interaction between drugs, targets, and diseases is complex. Our findings were mainly derived from online databases and network analysis; more cellular and animal experiments are needed to verify the predicted results of this study in the future. Researchers can first investigate drug-target interactions using computers and then validate the results through cell and animal experiments, which can more efficiently drive the clinical translation of drugs.

## Figures and Tables

**Table 1:** Key targets of escin against NP.

Gene name	Degree	Protein names
ALB	53	Albumin
SRC	52	Proto-oncogene tyrosine-protein kinase Src
JUN	43	Transcription factor AP-1
MMP9	41	Matrix metalloproteinase 9
IGF1	39	Insulin-like growth factor I
PTGS2	36	Prostaglandin G/H synthase 2
RHOA	36	Transforming protein RhoA
MAPK1	35	Mitogen-activated protein kinase 1
PPARG	33	Peroxisome proliferator-activated receptor gamma
IL2	30	Interleukin-2

**Table 2:** Binding interactions of escin against the core targets.

Targets	Binding interactions
PTGS2	Conventional hydrogen bonding-ASN A: 34, GLY A: 135, ASP A:158, GLY B:324; Alkyl and Pi-Alkyl-TYR A: 136, PHE B: 247, LYS B: 248
SRC	Conventional hydrogen bonding-SER A: 375, GLU A 520, ASN C:471; Alkyl and Pi-Alkyl-TYR-PHE A: 523, VAL C: 470; carbon hydrogen bond-ASP A: 368, THR A: 524, GLY C: 468
MMP9	Conventional hydrogen bonding-TYR B: 50, B: 52, B 179, GLY B: 100, PHE B: 107, PRO B: 180; Alkyl and Pi-Alkyl-LEU B: 44, ARG B 51, B: 95, LEU B: 187; carbon hydrogen bond-TYR B: 48, GLY B: 178
MAPK1	Conventional hydrogen bonding-MET A: 106, LYS A:149, SER A:151, ASN A:152; Alkyl-ALA A: 33, ARG A: 189; carbon hydrogen bond-GLU A: 31, ASP A: 109; Attractive charge-LYS A: 115; Pi-sigma-TYR A: 111

## Data Availability

The data used to support the findings of this study are available from the corresponding author on request.

## Supplementary Materials

Supplementary Materials Table S1: intersection target proteins of escin and NP. Table S2: the top 20 KEGG pathways. Figure S1: root mean square float (RMSF)-protein plots during of molecular dynamics simulation. (a) The RMSF of escin-PTGS2. (b) The RMSF of escin-SRC. (c) The RMSF of escin-MMP9. (d) The RMSF of escin-MAPK1. The plots display time (ns) on the *x*-axis and the RMSF (nm) on the *y*-axis. Figure S2: the radius of the protein complex's gyration (Rg). (a) Escin-PTGS2. (b) Escin-SRC. (c) Escin-MMP9. (d) Escin-MAPK1. The plots display time (ns) on the *x*-axis and the Rg (nm) on the *y*-axis.
