# Supplementary material for: Deciphering the Molecular Mechanism of Escin against Neuropathic Pain: A Network Pharmacology Study
Source: Evid Based Complement Alternat Med. 2023 Oct 16;2023:3734861. doi: 10.1155/2023/3734861 (PMC10593550; doi:10.1155/2023/3734861)
Supplement: Supplementary Materials — Table S1: intersection target proteins of escin and NP. Table S2: the top 20 KEGG pathways. Figure S1: root mean square float (RMSF)-protein plots during of molecular dynamics simulation. (a) The RMSF of escin-PTGS2. (b) The RMSF of escin-SRC. (c) The RMSF of escin-MMP9. (d) The RMSF of escin-MAPK1. The plots display time (ns) on the x-axis and the RMSF (nm) on the y-axis. Figure S2: the radius of the protein complex's gyration (Rg). (a) Escin-PTGS2. (b) Escin-SRC. (c) Escin-MMP9. (d) Escin-MAPK1. The plots display time (ns) on the x-axis and the Rg (nm) on the y-axis. [file 3734861.f1.docx]

**SUPPLEMENTARY MATERIALS**

**Deciphering the molecular mechanism of Escin against Neuropathic pain: A Network Pharmacology study**

Xi Li^a, 1^, Yating Wu^1^, Haoyan Wang^1^, Zaiqi Li^1^, Xian Ding^1^, Chongyang Dou^1^, Lin Hu^1^, [Guizhi Du](mailto:du_guizhi@yahoo.com)^2^, Guihua Wei^a, 1, *^

^1^ School of Life Science and Engineering, Southwest Jiaotong University, Chengdu, China

^2^ Department of Anesthesiology, Laboratory of Anesthesia and Critical Care Medicine, National-Local Joint Engineering Research Centre of Translational Medicine of Anesthesiology, West China Hospital, Sichuan University

lixixx@my.swjtu.edu.cn (X.L.); 2721735195@my.swjtu.edu.cn (Y.W.); 2022201014@my.swjtu.edu.cn (H.W.); zqli@my.swjtu.edu.cn (Z.L.); dingxian@my.swjtu.edu.cn (X.D.); dou@my.swjtu.edu.cn (C.D.); hulin.swjtu.edu.cn@my.swjtu.edu.cn (L.H.); duguizhi@scu.edu.cn (G.D.); weiguihua@home.swjtu.edu.cn (G.W.)

* Corresponding author: Guihua Wei. weiguihua@home.swjtu.edu.cn

^a^ Xi Li^1^, Guihua Wei^1^ contributed equally.

**Table S1** Intersection target proteins of escin and NP

| Targets | Uniprot ID | Protein names |
| --- | --- | --- |
| ACP3 | P15309 | Prostatic acid phosphatase |
| ADAM17 | P78536 | Disintegrin and metalloproteinase domain-containing protein 17 |
| ADCY1 | Q08828 | Adenylate cyclase type 1 |
| ADK | P55263 | Adenosine kinase |
| AKR1B1 | P15121 | Aldo-keto reductase family 1 member B1 |
| ALAD | P13716 | Delta-aminolevulinic acid dehydratase |
| ALB | P02768 | Albumin |
| ANG | P03950 | Angiogenin |
| APCS | P02743 | Serum amyloid P-component |
| APOA2 | P02652 | Apolipoprotein A-II |
| AR | P10275 | Androgen receptor |
| ARF1 | P84077 | ADP-ribosylation factor 1 |
| ATOX1 | O00244 | Copper transport protein ATOX1 |
| ATP1A1 | P05023 | Sodium/potassium-transporting ATPase subunit alpha-1 |
| BCHE | P06276 | Cholinesterase |
| BLVRB | P30043 | Flavin reductase |
| CA2 | P00918 | Carbonic anhydrase 2 |
| CCL5 | P13501 | C-C motif chemokine 5 |
| CDC42 | P60953 | Cell division control protein 42 homolog |
| CHIT1 | Q13231 | Chitotriosidase-1 |
| CMA1 | P23946 | Chymase |
| CTSB | P07858 | Cathepsin B |
| CTSF | Q9UBX1 | Cathepsin F |
| CTSS | P25774 | Cathepsin S |
| CYP2C9 | P11712 | Cytochrome P450 2C9 |
| EPHX2 | P34913 | Bifunctional epoxide hydrolase 2 |
| ESR1 | P03372 | Estrogen receptor |
| F2 | P00734 | Prothrombin |
| FECH | P22830 | Ferrochelatase, mitochondrial |
| GBA | P04062 | Lysosomal acid glucosylceramidase |
| GM2A | P17900 | Ganglioside GM2 activator |
| GSK3B | P49841 | Glycogen synthase kinase-3 beta |
| GSR | P00390 | Glutathione reductase, mitochondrial |
| HINT1 | P49773 | Adenosine 5'-monophosphoramidase HINT1 |
| HK1 | P19367 | Hexokinase-1 |
| HMGCR | P04035 | 3-hydroxy-3-methylglutaryl-coenzyme A reductase |
| IGF1 | P05019 | Insulin-like growth factor I |
| IGF1R | P08069 | Insulin-like growth factor 1 receptor |
| IKBKB | O14920 | Inhibitor of nuclear factor kappa-B kinase subunit beta |
| IL2 | P60568 | Interleukin-2 |
| INSR | P06213 | Insulin receptor |
| JAK2 | O60674 | Tyrosine-protein kinase JAK2 |
| JUN | P05412 | Transcription factor AP-1 |
| KIT | P10721 | Mast/stem cell growth factor receptor Kit |
| LYZ | P61626 | Lysozyme C |
| MAOB | P27338 | Amine oxidase |
| MAP2K1 | Q02750 | Dual specificity mitogen-activated protein kinase kinase 1 |
| MAPK1 | P28482 | Mitogen-activated protein kinase 1 |
| MAPK12 | P53778 | Mitogen-activated protein kinase 12 |
| MAPK14 | Q16539 | Mitogen-activated protein kinase 14 |
| MAPK8 | P45983 | Mitogen-activated protein kinase 8 |
| MIF | P14174 | Macrophage migration inhibitory factor |
| MMP1 | P03956 | Interstitial collagenase |
| MMP2 | P08253 | 72 kDa type IV collagenase |
| MMP8 | P22894 | Neutrophil collagenase |
| MMP9 | P14780 | Matrix metalloproteinase-9 |
| NOS2 | P35228 | Nitric oxide synthase, inducible |
| NR1H2 | P55055 | Oxysterols receptor LXR-beta |
| NR3C1 | P04150 | Glucocorticoid receptor |
| OPRK1 | P41145 | Kappa-type opioid receptor |
| PDE5A | O76074 | cGMP-specific 3',5'-cyclic phosphodiesterase |
| PDPK1 | O15530 | 3-phosphoinositide-dependent protein kinase 1 |
| PKLR | P30613 | Pyruvate kinase PKLR |
| PPARA | Q07869 | Peroxisome proliferator-activated receptor alpha |
| PPARG | P37231 | Peroxisome proliferator-activated receptor gamma |
| PRKACA | P17612 | cAMP-dependent protein kinase catalytic subunit alpha |
| PRKCA | P17252 | Protein kinase C alpha type |
| PRKCB | P05771 | Protein kinase C beta type |
| PRKCD | Q05655 | Protein kinase C delta type |
| PRKCE | Q02156 | Protein kinase C epsilon type |
| PRKCG | P05129 | Protein kinase C gamma type |
| PRKCH | P24723 | Protein kinase C eta type |
| PRKCQ | Q04759 | Protein kinase C theta type |
| PTGS2 | P35354 | Prostaglandin G/H synthase 2 |
| PTPN11 | Q06124 | Tyrosine-protein phosphatase non-receptor type 11 |
| REN | P00797 | Renin |
| RHOA | P61586 | Transforming protein RhoA |
| SELE | P16581 | E-selectin |
| SELP | P16109 | P-selectin |
| SHMT1 | P34896 | Serine hydroxymethyltransferase, cytosolic |
| SRC | P12931 | Proto-oncogene tyrosine-protein kinase Src |
| STS | P08842 | Steryl-sulfatase |
| TERT | O14746 | Telomerase reverse transcriptase |
| TLR9 | Q9NR96 | Toll-like receptor 9 |
| TRPV1 | Q8NER1 | Transient receptor potential cation channel subfamily V member 1 |
| TRPV4 | Q9HBA0 | Transient receptor potential cation channel subfamily V member 4 |
| TTPA | P49638 | Alpha-tocopherol transfer protein |
| TTR | P02766 | Transthyretin |
| VDR | P11473 | Vitamin D3 receptor |
| YARS1 | P54577 | Tyrosine--tRNA ligase, cytoplasmic |

**Table S2** The top 20 KEGG pathways.

| **Pathway ID** | **Description** | **pvalue** | **Relevant gene** |
| --- | --- | --- | --- |
| hsa04750 | Inflammatory mediator regulation of TRP channels | 2.09442E-13 | PRKCG, PRKCH, PRKCB, SRC, PRKCE, PRKCD, PRKCA, ADCY1, TRPV1, IGF1, MAPK14, MAPK12, MAPK8, TRPV4, PRKCQ, PRKACA |
| hsa04912 | GnRH signaling pathway | 2.41641E-11 | MAP2K1, JUN, PRKCB, SRC, MMP2, PRKCD, PRKCA, ADCY1, MAPK14, MAPK12, CDC42, MAPK8, MAPK1, PRKACA |
| hsa05205 | Proteoglycans in cancer | 7.79255E-11 | PRKCG, MAP2K1, PRKCB, PDPK1, SRC, MMP2, PRKCA, PTPN11, IGF1, MAPK14, ESR1, MMP9, RHOA, MAPK12, IGF1R, CDC42, MAPK1, PRKACA |
| hsa05200 | Pathways in cancer | 3.15375E-10 | PRKCG, GSK3B, MAP2K1, JUN, NOS2, PRKCB, MMP1, MMP2, PRKCA, ADCY1, IGF1, PTGS2, MMP9, RHOA, IGF1R, CDC42, IKBKB, AR, MAPK8, KIT, MAPK1, PPARG, PRKACA |
| hsa04722 | Neurotrophin signaling pathway | 1.01063E-08 | GSK3B, MAP2K1, JUN, PDPK1, PRKCD, PTPN11, MAPK14, RHOA, MAPK12, CDC42, IKBKB, MAPK8, MAPK1 |
| hsa04660 | T cell receptor signaling pathway | 1.6018E-08 | CDC42, IKBKB, GSK3B, MAP2K1, JUN, PDPK1, MAPK1, PRKCQ, MAPK14, RHOA, IL2, MAPK12 |
| hsa04370 | VEGF signaling pathway | 2.61505E-08 | CDC42, PRKCG, MAP2K1, SRC, PRKCB, MAPK1, PRKCA, MAPK14, PTGS2, MAPK12 |
| hsa05120 | Epithelial cell signaling in Helicobacter pylori infection | 6.09546E-08 | CDC42, IKBKB, ADAM17, JUN, MAPK8, SRC, CCL5, PTPN11, MAPK14, MAPK12 |
| hsa04270 | Vascular smooth muscle contraction | 8.37631E-08 | PRKCG, MAP2K1, PRKCH, PRKCB, PRKCE, PRKCD, MAPK1, PRKCQ, PRKCA, ADCY1, PRKACA, RHOA |
| hsa04015 | Rap1 signaling pathway | 1.00757E-07 | PRKCG, MAP2K1, PRKCB, SRC, INSR, PRKCA, ADCY1, IGF1, MAPK14, RHOA, MAPK12, IGF1R, CDC42, KIT, MAPK1 |
| hsa04014 | Ras signaling pathway | 2.51083E-07 | PRKCG, MAP2K1, PRKCB, INSR, PRKCA, PTPN11, IGF1, RHOA, IGF1R, CDC42, IKBKB, MAPK8, KIT, MAPK1, PRKACA |
| hsa04960 | Aldosterone-regulated sodium reabsorption | 2.51366E-07 | PRKCG, PDPK1, PRKCB, INSR, MAPK1, PRKCA, IGF1, ATP1A1 |
| hsa05142 | Chagas disease (American trypanosomiasis) | 2.75227E-07 | IKBKB, JUN, MAPK8, NOS2, CCL5, TLR9, MAPK1, ADCY1, MAPK14, IL2, MAPK12 |
| hsa04668 | TNF signaling pathway | 3.60263E-07 | IKBKB, MAP2K1, JUN, MAPK8, CCL5, MAPK1, MAPK14, PTGS2, SELE, MMP9, MAPK12 |
| hsa04931 | Insulin resistance | 3.93329E-07 | IKBKB, GSK3B, MAPK8, PDPK1, NR1H2, INSR, PRKCE, PRKCD, PRKCQ, PTPN11, PPARA |
| hsa04510 | Focal adhesion | 5.7379E-07 | PRKCG, GSK3B, MAP2K1, JUN, PRKCB, PDPK1, SRC, PRKCA, IGF1, RHOA, IGF1R, CDC42, MAPK8, MAPK1 |
| hsa04919 | Thyroid hormone signaling pathway | 7.09163E-07 | PRKCG, GSK3B, MAP2K1, PDPK1, SRC, PRKCB, MAPK1, PRKCA, ATP1A1, PRKACA, ESR1 |
| hsa04071 | Sphingolipid signaling pathway | 1.05378E-06 | PRKCG, MAP2K1, MAPK8, PDPK1, PRKCB, PRKCE, MAPK1, PRKCA, MAPK14, RHOA, MAPK12 |
| hsa04930 | Type II diabetes mellitus | 1.10638E-06 | IKBKB, MAPK8, PKLR, INSR, PRKCE, PRKCD, MAPK1, HK1 |
| hsa04917 | Prolactin signaling pathway | 1.38067E-06 | GSK3B, MAP2K1, MAPK8, SRC, MAPK1, JAK2, MAPK14, ESR1, MAPK12 |


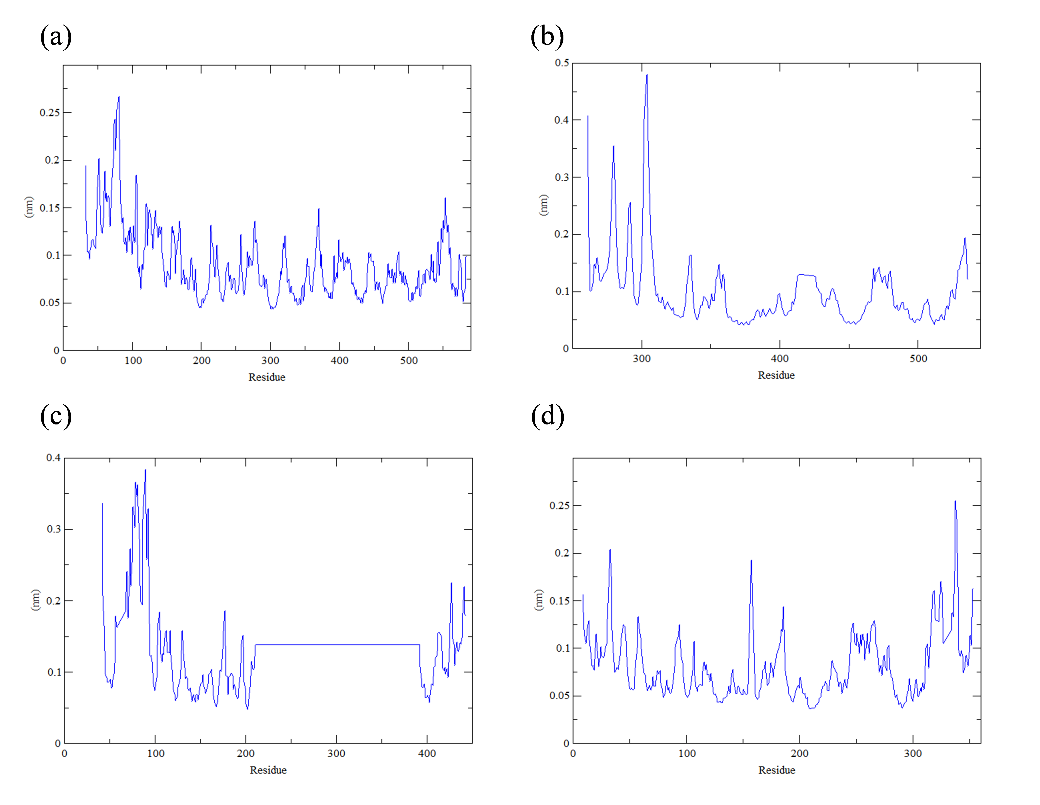


**Fig. S1.** Root Mean Square Float (RMSF)-Protein plots during of molecular dynamics simulation. (a) The RMSF of escin-PTGS2. (b) The RMSF of escin-SRC. (c) The RMSF of escin-MMP9. (d) The RMSF of escin-MAPK1. The plots display time (ns) on the x- axis and the RMSF (nm) on the y-axis.


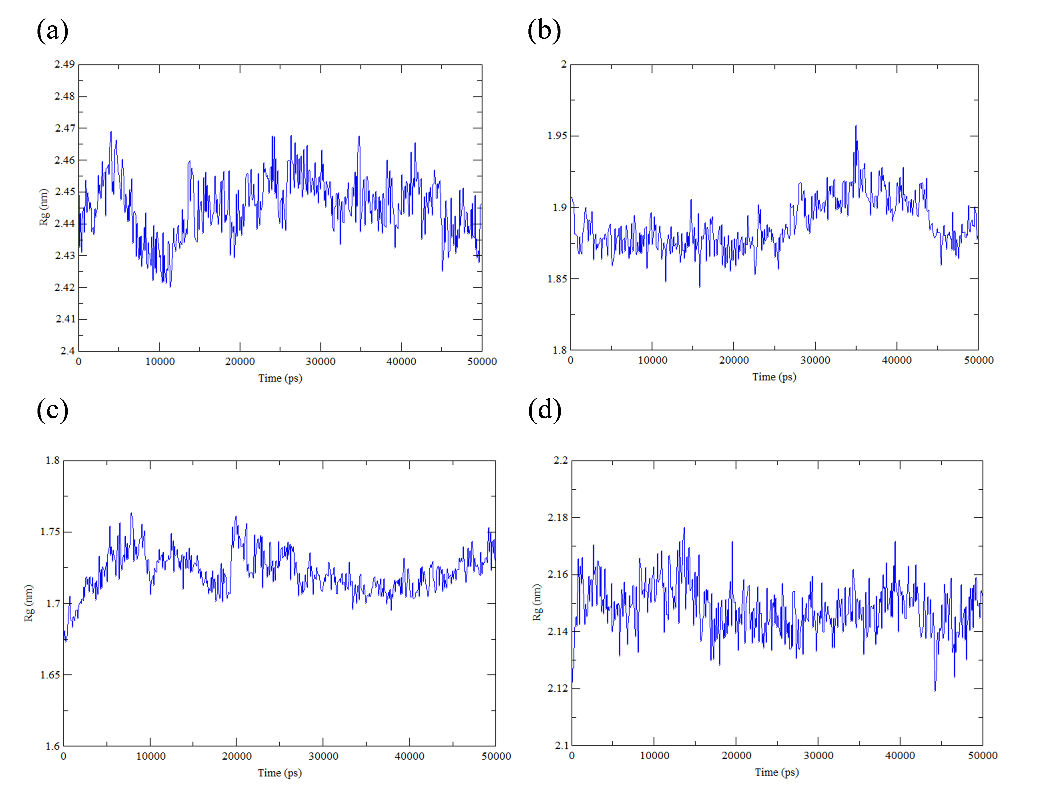


**Fig. S2.** The radius of the protein complex’s gyration (Rg). (a) Escin-PTGS2. (b) Escin-SRC. (c) Escin-MMP9. (d) Escin-MAPK1. The plots display time (ns) on the x- axis and the and the Rg (nm) on the y-axis.
